# Young People’s Experiences Using an On-Demand Mobile Health Sexual and Reproductive Health Text Message Intervention in Kenya: Qualitative Study

**DOI:** 10.2196/19109

**Published:** 2021-01-15

**Authors:** Jefferson Mwaisaka, Lianne Gonsalves, Mary Thiongo, Michael Waithaka, Hellen Sidha, Otieno Alfred, Carol Mukiira, Peter Gichangi

**Affiliations:** 1 International Centre for Reproductive Health, Kenya Mombasa Kenya; 2 Department of Population Family and Reproductive Health College of Health Sciences University of Ghana Accra Ghana; 3 UNDP-UNFPA-UNICEF-WHO-World Bank Special Programme of Research, Development and Research Training in Human Reproduction (HRP), Department of Sexual and Reproductive Health and Research World Health Organization Geneva Switzerland; 4 Swiss Tropical and Public Health Institute Basel Switzerland; 5 University of Basel Basel Switzerland; 6 National Council for Populations and Development Kenya Nairobi Kenya; 7 Population Studies and Research Institute University of Nairobi Nairobi Kenya; 8 African Institute for Development Policy Nairobi Kenya; 9 Ghent University Ghent Belgium; 10 Technical University of Mombasa Mombasa Kenya

**Keywords:** mHealth, mobile phones, sexual and reproductive health, Kenya

## Abstract

**Background:**

Digital health usability assessments can help explain how well mobile health (mHealth) apps targeting young people with sexual and reproductive health (SRH) information performed and whether the intended purpose was achieved. However, few digital health assessments have been conducted to evaluate young people’s perceptions regarding mHealth system interactions and content relevance on a wide range of SRH topics. In addition, the majority of randomized controlled trials (RCTs) have focused on push messaging platforms; therefore, the mHealth field lacks sufficient RCTs investigating on-demand mHealth SRH platforms.

**Objective:**

The objective of this study was to explore young people’s experiences using an on-demand SRH mHealth platform in Kenya.

**Methods:**

We used qualitative data related to the usability of an mHealth platform, Adolescent/Youth Reproductive Mobile Access and Delivery Initiatives for Love and Life Outcome (ARMADILLO), collected at the end of the intervention period. A total of 30 in-depth interviews (IDIs) were held with the intervention participants (15 women and 15 men) to elicit their experiences, opinions, and perspectives on the design and content of the ARMADILLO platform. The study participants were randomly selected from a list of intervention arm participants to participate in the IDIs. The interviews were later transcribed verbatim, translated into English, and coded and analyzed thematically using NVivo version 12 software (QSR International).

**Results:**

Respondents reported varied user experiences and levels of satisfaction, ranging from ease of use by the majority of the respondents to systematic frustrations that prevented some participants from progressing to other stages. Interesting features of the mHealth platform included the immediate response participants received when requesting messages, weekly remunerated quizzes, and perceived *ability* of educative and informative content and messages to change behaviors. Proposed enhancements to the platform included revising some concepts and words for easy understanding and increasing the interactivity of the platform, whereby young people could seek clarity when they came across difficult terms or had additional questions about the information they received.

**Conclusions:**

The importance of understanding the range of health literacy and technological variations when dealing with young people cannot be overemphasized. Young people, as mHealth end users, must be considered throughout intervention development to achieve optimum functionality. In addition, young people targeted with mHealth SRH interventions must be sensitized to the interactions on mHealth platforms or any other digital health apps if implemented in a nonresearch setting for optimal use by the targeted audience.

## Introduction

### Background

Globally, sexual and reproductive health (SRH) programs use mobile phones as communication platforms to reach out to young people aged between 10 and 24 years with information and services on a wide range of SRH topics [1]. The proliferation of mobile phones and the significant advancements in wireless technologies provide innovative ways to deliver health information to young people [2]. In addition, the flexibility, accessibility, confidentiality, and convenient nature of mobile phones make them appealing to young people seeking sensitive SRH information.

Assessing the usability and acceptability of various mobile health (mHealth) interventions designed to provide factual SRH information to adolescents and young people is an essential step in designing and implementing sustainable, appealing mHealth programs [3]. The World Health Organization (WHO) indicates that *usability* determines whether the mHealth intervention can be used as intended by the users by focusing on the quality of interaction between the user and the technology [4]. Usability assessments can be used to identify needs, design, develop, implement, and evaluate appropriate and effective mHealth interventions. Usability assessments involving young people as the targeted users can help explain how well the mHealth app functioned and whether the platform achieved its intended purpose [5].

Several studies have explored the potential of pushed text messaging to improve knowledge and affect SRH behavior change among young people and hard-to-reach populations [1,6-9]. However, there has been less research on interactive on-demand systems where health information is triggered by users’ requests, traditionally via SMS menus [10]. One enabler of mHealth adoption in developing countries such as Kenya is the interactive interface feature of health apps [11]. Interactive mHealth programs have been found to be feasible, acceptable, and potentially effective in supporting behavior change such as smoking cessation [12] and depressive symptoms among individuals with spinal cord injury [13]. In Kenya, interactive on-demand 2-way mHealth interventions have been shown to be superior to 1-way text message interventions, particularly for improving medication adherence among HIV-positive individuals; however, a significant knowledge gap still exists as to why on-demand 2-way mHealth interventions are deemed superior [14,15]. This could be because of inadequate formal usability and acceptability assessments to compare the 2 sets of mHealth delivery models. This lack of insight may contribute to future challenges such as integrating mHealth into health services and the adoption of mHealth interventions by policy makers for scale-up purposes [14]. In addition, existing on-demand mHealth apps in Kenya such as *Daktari popote*, meaning *Doctor anywhere*, *Hello Doctor*, and MedAfrica, which allow users to search and filter health information and locate doctors and hospitals, are largely internet based and would require users to have smartphones to access health services [16].

Specifically, for mHealth text message interventions targeting adolescents and young people, there are very few, if any, formal usability and acceptability assessments of interactive and on-demand mHealth SRH interventions, particularly in low-income countries [17]. The Mobile for Reproductive Health (m4RH) program is a unique program designed to expand family planning information, albeit for the general public, which is known to have been evaluated. The evaluation of the m4RH presented user satisfaction information with respect to the mHealth platform’s contents [18,19]; however, this evaluation did not capture the complete usability of the mHealth system, including user interactions and how effective the interactive mHealth platform was perceived by the users. For this reason, there is still a need for evaluations, particularly for those mHealth interventions designed to improve SRH knowledge and service uptake among young people, to focus on understanding targeted real users’ interactions. Such assessments can significantly benefit mHealth developers, users, and policy makers. The expected functionality of mHealth apps and recommendations gathered from such assessments will provide helpful guides for improving the usability of mHealth apps targeting young people.

### Study Objectives

This study sought to evaluate the usability of an interactive on-demand mHealth platform aimed at providing SRH information to young people in Kenya. Specifically, the assessment determined whether and to what extent young people found the digital intervention user friendly and useful by examining their experiences and perceptions regarding the system’s design, ease of interaction, and platform contents.

## Methods

### Overview

This usability assessment was nested within the broader Adolescent/Youth Reproductive Mobile Access and Delivery Initiatives for Love and Life Outcome (ARMADILLO) study. ARMADILLO was a 3-arm (*intervention, control,* and *contact*) individual randomized controlled trial (RCT; International Standard Randomized Controlled Trial Number [ISRCTN]: 85156148) aimed at developing and evaluating an on-demand system for young people to access and receive SRH information through SMS. The trial has been described in detail elsewhere [20]. In brief, the larger mHealth (ARMADILLO) study was conducted in Kwale County of Kenya, between January and July 2018, and with young male and female study participants aged 18 to 24 years. Compared with other counties in the coastal region of Kenya, Kwale County is among the top 2 counties with the highest prevalence of teenage pregnancies (24%), which is higher than both regional and national averages reported at 20.8% and 18%, respectively [21]. In addition, according to the Kenya Demographic and Health Survey of 2014, Kwale County had the lowest median age at first sexual intercourse among women, 16.6 years in the entire coastal region of Kenya, and the fifth lowest nationally [21]. Moreover, the county had a lower contraceptive prevalence rate (CPR) of 38.2% compared to the national CPR of 53.2% [21].

### Participants’ Recruitment

The ARMADILLO study used household-based surveys and multistage random sampling to recruit participants for the main study. We used census data obtained from the Kenya National Bureau of Statistics to identify and enumerate all households in the study zone. This was followed by a census of households with eligible study participants. A list of potential participants to be sampled was then randomly generated using a computer-based random number generator. The research team then made a second visit to the selected households to inform eligible participants about the study and seek consent from them. A total of 740 young men and women aged 18 to 24 years were selected for the study. This sample provided 80% power to detect a 10% change in mean number of myths believed by young people from baseline to end line, assuming that the baseline level of belief was 0.55, with an assumed SD of 0.30, accounting for a dropout rate of 20% [20]. To minimize contamination, only 1 eligible youth was selected from each household. This was followed by randomly allocating participants into intervention, contact, or control groups using a computer-based algorithm. The allocation followed a 1:1:1 ratio; this allocation was overseen by a member of the research team who did not interact with the study participants. After the arm allocation, the participants’ 7-week interaction with the relevant arm began the following day [20].

### Intervention Procedure

**Figure 1:**
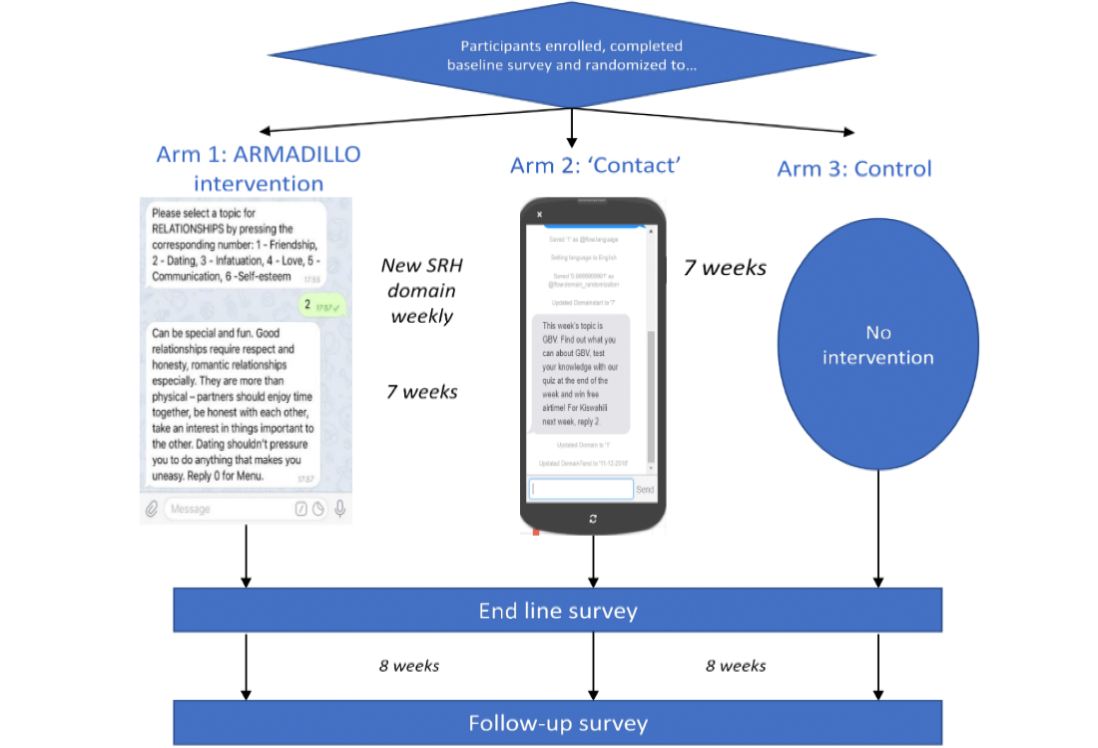
Adolescent/Youth Reproductive Mobile Access and Delivery Initiatives for Love and Life Outcome trial diagram. ARMADILLO: Adolescent/Youth Reproductive Mobile Access and Delivery Initiatives for Love and Life Outcome; SRH: sexual and reproductive health.

The intervention period was from January 20, 2018, to March 10, 2018, lasting 7 weeks with outcome assessments conducted at baseline and end line. The trial’s primary outcome was to assess the ability of on-demand SRH information delivered via SMS to dispel myths and misconceptions around contraception. Each week during the intervention period (Figure 1), participants randomized to the intervention arm received a given SRH domain pushed to their phones and subsequently requested information (subdomains) related to that particular topic. The contact arm participants received weekly topics only and were instructed to learn on their own. The control arm received no messages. This paper highlights the experiences of only intervention arm participants.

### Intervention Arm Design

**Figure 2:**
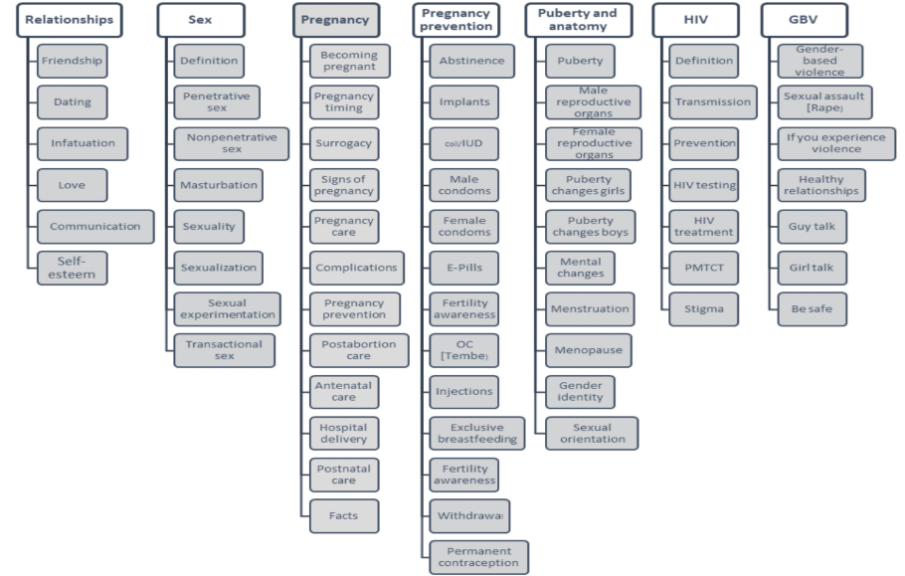
Adolescent/Youth Reproductive Mobile Access and Delivery Initiatives for Love and Life Outcome intervention arm architecture showing domains and subdomains. ARMADILLO: Adolescent/Youth Reproductive Mobile Access and Delivery Initiatives for Love and Life Outcome. Pregnancy Prevention. GBV: gender-based violence; IUD: intrauterine device; E-pills: emergency contraceptive pills; OC: oral contraceptives; PMTCT: prevention of mother to child transmission of HIV.

Following enrollment into the system, the intervention arm participants received an introductory SMS followed by a second SMS asking them to specify whether they would prefer messages in English or Swahili (language preference could be changed throughout the intervention). They then received their first, randomly selected weekly domain, marking the start of their 7-week intervention period. The platform presented the intervention arm participants with an SMS menu of various subtopics from the weekly domain (Figure 2), and the intervention participants were asked to select a number to learn more about the selected subdomain.

Interaction with the platform incurred no charges from the participants, and all costs were billed to the study. Intervention arms participants were prompted to engage with weekly quizzes, after which free airtime of US $ 0.50 was credited to their phones by the study irrespective of whether their responses were correct or not. Per ethics requirements, participants were periodically reminded that they could opt out of the study by sending the word *STOP* to the short code, given as 21438. All interactions with the system were free for participants, with any SMS costs reverse billed to the study.

**Figure 3:**
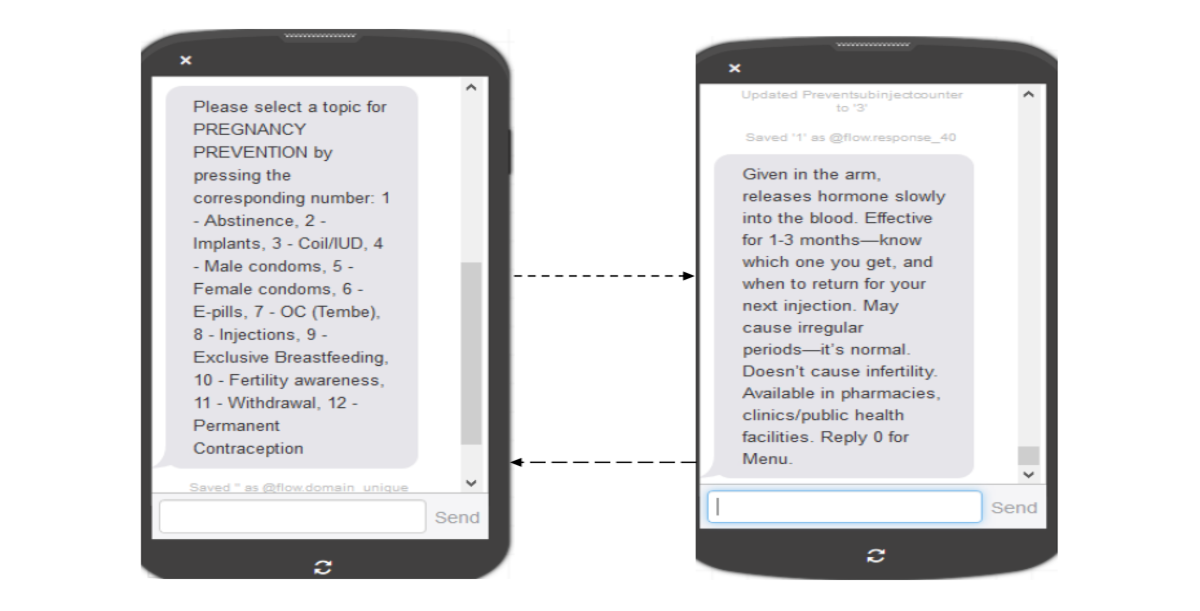
Example Adolescent/Youth Reproductive Mobile Access and Delivery Initiatives for Love and Life Outcome subdomain menu with sample ‘injection (#8) message requested’ (English version).

Participants could request a given subdomain using a number-based menu (Figure 3)—each subdomain request resulted in 1 to 3 SMS worth of content pushed to young people’s mobile phones. The number-based menu was selected, as it is commonly used by mobile phone users in Kenya to purchase mobile airtime and/or internet bundles directly from their service provider and for remote mobile money transfers. Finally, weekly domains were sent with an opt-out option where participants could unsubscribe from the platform at will.

### Data Collection

Qualitative data related to the ARMADILLO platform were collected at the end of the 7-week intervention period of the study. The in-depth interviews (IDIs) were held between April 2018 and June 2018. Out of 206 intervention arm participants who did not drop out of the study and completed both baseline and end line assessments, 30 intervention arm participants (15 females and 15 males) were randomly selected from a list of intervention arm participants to participate in an IDI. The concept of saturation in qualitative methods limiting the number of IDIs to between 25 and 30 participants required to reach thematic saturation [22] was employed. The IDIs sought to elicit participants’ experiences, opinions, and perspectives on the design and content of the ARMADILLO platform. Following randomization, the study team made phone calls to the selected intervention arm participants to check on their availability. If one was not available, another participant was randomly selected until a predetermined sample size of 30 intervention respondents was achieved.

A total of 4 interviewers (2 men and 2 women) were trained to conduct the interviews and were provided with a semistructured interview guide. All interviewers had university-level education, 2 were public health experts, whereas the other 2 were social scientists. The interview guides were developed by the research team; the interview questions determined by the research team were reviewed by experts from the International Centre for Reproductive Health-Kenya. After the review of the interview questions, the guides were piloted with young people aged 18 to 24 years from a neighboring county. Suggested changes arising from piloting were reported to the research team and appropriate modifications were made. The guide content included questions on experiences while interacting with the platform; the typical question was “Tell me about your experience interacting with the platform, how was it? Easy? Difficult?” Other questions included what participants liked and/or disliked about the ARMADILLO platform and participants’ views about the ARMADILLO domains, including their relevance and comprehensibility as well as suggestions for improvements.

All interviews were conducted in person at a private room in a local drop-in center (a nongovernmental health facility where young people and key populations receive comprehensive package of SRH services). Each data collector interviewed participants of the same sex. All interviews were conducted in Swahili, and following the consent of the respondents, all discussions were audio recorded.

The interviews were later transcribed verbatim, translated into English, and coded and analyzed thematically using NVivo (QSR International) version 12 software. Two researchers read through all the 30 transcripts independently; repeated readings of all the transcripts to search for meanings and patterns were done to further familiarize ourselves with the data. This was followed by code development from the data where the researchers identified important statements from the transcripts and attached a label to them to guide in developing themes. Four research meetings were held throughout the coding process to discuss emerging ideas arising from the coded data sets. These meetings helped in theme development. Our code development and thematic analysis was data driven, and we used inductive coding to create codes based on the responses of the study participants. Our analysis was shaped by the frequency of similar codes and recurrent ideas based on similarities in the data. Following theme development, the researchers reviewed the coded data extracts from each theme for coherence and consistency until all the researchers were satisfied that the data have been logically presented in a useful manner. A framework thematic analysis approach [23] was used to develop the themes. The definition of *usability of digital health interventions* by the WHO informed the coding process [4]. Our indicators of analysis, therefore, sought to determine whether the mHealth intervention worked, in that, it was easy to interact with (user friendly), and whether young people found the platform useful. Our analysis and findings are presented in accordance with the Consolidated Criteria for Reporting Qualitative Research [24].

Ethical clearance was obtained from the WHO Institutional Review Board (Protocol WHO A65892 core) and the Kenyatta National Hospital-University of Nairobi Ethics and Review Committee (KNH-UoN-ERC P550/09/2014).

## Results

### Demographic Characteristics and Phone Ownership of Study Participants

An equal number of men and women (15 each) were interviewed in this study. The mean age for all participants was 21.13 years, with an SD of 1.96. A greater proportion of women 33% (5/15) compared with men 27% (4/15) had postsecondary education (Table 1). More men 40% (6/15) had secondary school education as their highest education level compared with their women counterparts (4/15, 27%). More women 87% (13/15) than men (8/15, 53%) owned smartphones. This study also established that more than half of the study participants had their current phones for more than a year men, 53% (8/15) and women, 80% (12/15), as shown in Table 1.

Qualitative findings from this study are categorized into following 3 themes:

1. High and low points of an on-demand system
2. Navigating the system’s supportive features: what worked and what didn’t?
3. SRH content relevance and comprehension: Was it useful?

**Table 1:** Sociodemographic characteristics of study participants by sex (N=30).

Characteristic		Youth, men			Youth, women		
		Age 18-19 years (n=3, 20%)	Age 20-24 years (n=12, 80%)	Total (n=15, 100%)	Age 18-19 years (n=4, 27%)	Age 20-24 years (n=11, 73%)	Total (n=15, 100%)
**Highest school level, n (%)**							
	Never attended school	N/A^a^	1 (8.3)	1 (6.6)	1 (25.0)	N/A	1 (6.7)
	Primary school	2 (66.7)	2 (16.7)	4 (26.7)	2 (50.0)	3 (27.3)	5 (33.3)
	Secondary school	N/A	6 (50.0)	6 (40.0)	1 (25.0)	3 (27.3)	4 (26.7)
	Postsecondary school	1 (33.3)	3 (25.0)	4 (26.7)	N/A	5 (45.4)	5 (33.3)
**Relationship status, n (%)**							
	Single	2 (66.7)	6 (50.0)	8 (53.3)	2 (50.0)	4 (36.4)	6 (40.0)
	Dating or friends with benefits	1 (33.3)	5 (41.7)	6 (40.0)	N/A	7 (63.6)	7 (46.7)
	Married or engaged	N/A	1 (8.3)	1 (6.7)	2 (50.0)	N/A	2 (13.3)
**Have a child, n (%)**							
	Yes	N/A	2 (16.7)	2 (13.3)	N/A	N/A	N/A
	No	3 (100)	10 (83.3)	13 (86.7)	4 (100)	11 (100)	15 (100)
**Phone type, n (%)**							
	Analog phone (only for calls and text messages)	N/A	3 (25.0)	3 (20.0)	N/A	1 (9.1)	1 (6.7)
	Multimedia phone (has a camera and MP3^b^ player but does not have the internet or apps)	1 (33.3)	3 (25.0)	4 (26.7)	1 (25.0)	N/A	1 (6.7)
	Smartphone (has internet and apps)	2 (66.7)	6 (50.0)	8 (53.3)	3 (75.0)	10 (90.9)	13 (86.6)
**Duration of phone ownership, n (%)**							
	Less than 1 month	N/A	1 (8.3)	1 (6.7)	1 (25.0)	N/A	1 (6.7)
	1-3 months	1 (33.3)	N/A	1 (6.7)	N/A	N/A	N/A
	3-6 months	N/A	4 (33.3)	4 (26.7)	N/A	N/A	N/A
	6-12 months	1 (33.3)	N/A	1 (6.7)	1 (25.0)	1 (9.1)	2 (13.3)
	More than one year	1 (33.3)	7 (58.4)	8 (53.3)	2 (50.0)	10 (90.9)	12 (80.0)

^a^N/A: not applicable.

^b^MP3: MPEG audio layer-3.

### High and Low Points of an On-Demand System

On-demand systems are common in Kenya. When asked about their experiences interacting with the ARMADILLO platform, a majority of the IDI participants reported that the platform’s procedures were easy to follow and that they were able to navigate through the platform on their own. Both female and male participants reported positive user experiences and were generally able to move through the steps without any difficulties. The similarity of the mHealth platform with other on-demand mobile services offered by the majority of the mobile service providers in Kenya to buy airtime, pay bills, and order for other services was largely associated with the ease of platform use:

I can say it was easy. ... I reached the messages by following the instructions that were given, they were simple instructions to follow then get to a conclusion

23-year-old male youth

When I received the messages, I used to follow the directions like they were sending let’s say from number 1-12, so I went through them one by one ... I used to send one after the other, when I send the first one, they bring me the second one until I finished. And they used to respond to what I was asking them

21-year-old female youth

However, the ease of use was not entirely uniform. Reported restrictions included repeat instructions to participants, preventing them from progressing to other stages. A few users reported difficulties completing some functions, such as getting back to the menu by selecting *0*. This was because some participants reported that they were used to the feature (reply *0* for menu) being called *home* in other apps as opposed to menu, whereas others preferred it to be called the *go back* feature. In addition, a few participants mistakenly put a number not available on the menu and kept getting an *I don’t understand your selection* response. When designing this mHealth platform, such challenges did not seem straightforward, probably based on the assumption that the navigation process was clear and that young people were adept at using mobile phones:

I experienced difficulties two times since every time I responded I could see those are the same questions being asked and when I reply am told they do not understand my response, until the third time that is when I entered one option and I realized that they were topics, I did not know and understand the first time.

19-year-old female youth

That said, generally, when participants were unclear, they sought assistance from their peers in the study. Despite being informed that they could reach out to the study team in case of any technical challenges, young people in this study tended to be far more comfortable seeking help from their peers than from the research team. This speaks to the component of peer-to-peer learning among young people, typical of how they rely on their peers for advice. Although no female participants reported reaching out to their peers for technical support, male participants in this study reported some challenges in progressing through the system and preferred to reach out to their friends:

First, I did not know how to go about it, but I got advice from someone else who instructed me and told me how it was and how to use it.

22-year-old male youth

I inquired from a friend, I did not know how to go about it and there was someone called XXX or I am not supposed to mention him ... I followed him and he explained to me.

20-year-old male youth

A feature of on-demand systems is the ability to receive targeted information on a topic based on a user’s interest *in the moment.* Respondents appreciated the *immediate response* they received from the system when requesting a message from a subdomain. Participants did not indicate any long breaks or delays that would have discouraged them from interacting with the platform. Almost all participants interviewed provided this positive comment regarding the ARMADILLO platform. The capacity for real-time feedback resulted in some motivation by most users, which made them continue to interact with the system. Perhaps this is an interesting observation. The impatient nature of young people was clearly displayed; mHealth platforms, therefore, need to provide real-time support to increase their usability when targeting young people:

... when you request for something there was immediate feedback ... and it’s like there is somebody attending to you, there were no delays.

18-year-old male youth

What I liked most about ARMADILLO was the information they shared, it was so helpful and there were no delays. Anyone who wanted to get the whole information would press a number and one would get it instantly.

23-year-old male youth

A limitation of on-demand systems is that, similar to push systems, there is no ability to engage further for questions and clarifications beyond what the developers have predefined. This *static* nature caused some frustration among participants, who wanted additional clarity when they came across difficult terms or had additional questions about the information received. This concern came from both male and female participants; young people wanted to learn more about SRH issues, particularly on pregnancy prevention methods. This finding suggests that a platform allowing young people to ask SRH questions via text messages and being responded to by trained SRH experts would appeal more to young people and contribute to improved mHealth interactions:

I do not know about the others if they received, or maybe they had people to ask who understood but as for me I had no one to ask, I would give an answer to get the credit but ... I just want to request if one was wrong then they should be able to direct them into getting the right answer, because one does not know what the right or wrong answer is since whether right or wrong one still received credit

21-year-old female youth

There was a time I received some information about something, but I was not contented because I did not understand ... there was an issue about family planning, using of pills and injection ... There are some that I understood, and there are others that I needed more explanation, not everything that one reads they get to understand.

22-year-old male youth

### Navigating the System’s Supportive Features: What Worked and What Didn’t?

The weekly, remunerated quizzes (initially developed to encourage participants to engage with the system and motivate them to participate) were well received by study participants. Some participants were explicit about being motivated by the free airtime to interact with the system. However, others felt that the weekly quizzes were a good feature that positively challenged their knowledge. The majority of participants expressed their excitement with the weekly quizzes terming them as learning motivators that interested and made them want to engage more with the platform:

I liked the questions part because they promote you with credit at the end of answering and also, they give you a duration if you are free, they ask you questions then after answering they send you credit

23-year-old female youth

What I liked was when I was asked the weekly questions, some questions I did not know about them, while others I did; so, I used to think if I was able to answer them, then am good ... That challenge was what I liked.

21-year-old female youth

However, 2 additional design features were a source of confusion for the participants. First, a number of participants reported that they ignored the first messages, as they did not recognize the ARMADILLO short code (21438) and thought that the messages were spam, sent every so often by local service providers. Although short codes are the preferred channels for mHealth bulk SMS text messaging, the skepticism tendency displayed by young people implies that the proliferation of SMS services and spam messages could threaten the mHealth platform’s acceptability and usability, particularly by young people who appeared cautious when interacting with unverified sources. For this study, had the messages been sent with an *ARMADILLO* name, chances are that participants would not have ignored their first messages:

When I received the message I did not know, any time I would receive the messages and think they are from ... service provider ...and ignored them only to realize they were from ARMADILLO.

24-year-old male youth

I was shocked ... I have never gotten such questions or messages, ... I was like who are these people? Of all the people why did they choose me... as in to ask me because ... I did not know they [people sending the messages] were the ones like the same with ARMADILLO.

18-year-old female youth

Second, participants were periodically reminded that they could opt out of the system (and study) by sending *STOP* back to the short code. Unfortunately, some participants thought that this opt-out option was similar to *periodic* unsubscribing from mobile provider services, which allow users to opt out (eg, when not interested in something) but also opt *in* later on. However, to comply with research ethics requirements, opting out meant dropping out of the study altogether:

For example, if I wanted to dismiss myself from the group it was a bit of a challenge and I would keep asking myself... for example if I dismiss myself from the system, I did not know whether I would continue getting the messages later on or not, so I did not know how to go about that.

22-year-old female youth

### SRH Content Relevance and Comprehension: Was It Useful?

Participants generally felt that the ARMADILLO contents were relevant to them and people of their age. Messages were described as being educative and informative. Not only did the participants receive new information but they were also conveyed the information with clarity, devoid of the shame mostly associated with SRH talk. SRH knowledge, candidly described, resonated with the young participants of ARMADILLO. Topics touching on sex, HIV, and pregnancy were the most mentioned domains from the platform by both male and female participants:

...they have helped me to overcome some myths, be open minded, you know...as in .... There are myths like, for instance that young girls cannot get pregnant or get AIDs ... There are also those that say when one engages in sex for the first time, she cannot get pregnant. Something of that sort makes someone wise; you can understand that it is important to use protection when engaging in sex

21-year-old male youth

What I liked most is that most issues discussed were issues that were not openly discussed as they are considered shameful, in fact in this generation it has been difficult for us to sit with our parents and be explained to that ‘nowadays you are supposed to be this and that way’... when I came across ARMADILLO, I got the opportunity that I have been missing ... I have even known how to avoid certain things.

23-year-old male youth

Anecdotally, some messages were purported to have inspired intent to change behavior. Participants reported going for HIV testing and deciding to use condoms as a result of the messages:

The other ones like HIV testing...as in there is a time I had stayed long without testing, When I read those messages, I got the courage to go and get tested because it had been long.

21-year-old female youth

... to prevent myself, as in health wise, to prevent myself by using a condom that is what made me happy, I read about how I can help myself when I meet a woman or how to ensure she does not get pregnant.

20-year-old male youth

Although a majority of the participants reported satisfaction with the platform’s use of language (in both English and Swahili), a few indicated that certain concepts and words used were difficult to understand. The language challenge was largely reported by the female participants. Unlike their male counterparts who reported reaching out to their friends when they experienced challenges navigating the platform, the female participants chose to use the web in search of explanations, whereas others chose to keep their uncertainties to themselves:

At times the English used was hard and it became complicated, but I would use google for the meaning if I did not understand, because their English was deeper, so I just ‘googled’ the meaning and I go through it.

18-year-old female youth

Difficult was the family planning [apart from condoms and emergency pills], the subdomains about family planning [pregnancy prevention]; even though I requested, [when] they gave me the messages I was like ... I am not understanding anything.

21-year-old female youth

## Discussion

### Principal Findings

In this qualitative study, we describe young peoples’ experiences using an on-demand mHealth intervention and thoughts about the content. Young participants in the ARMADILLO trial had a variety of experiences when interacting with the mHealth platform. Once a weekly domain was opened, the majority of participants reported enjoying the freedom to select the subdomain they wished to read at their convenience. However, although designed for intuitive engagement, the ease of use as envisioned by the study was not uniform. A few users reported getting confused by the aspects of the system. Others expressed frustration when they reached the end of a message and were left with additional questions. Weekly quizzes aimed at motivating the study participants to interact with the system were appreciated. The guaranteed mobile credit was probably responsible for a fair amount of participants’ enthusiasm. Misunderstandings about the system’s *stop* feature and short code caused additional confusion. Finally, the content itself was widely appreciated and understood by most participants; a few, however, cited comprehension difficulty.

### mHealth Situation in Kenya

Most of the myriad mHealth projects in Kenya (both on-demand and pushed services) are rarely evaluated to assess their usability by the targeted audience [17]. One exception is an evaluation of the m4RH program, which was designed to expand the access to family planning information for the general public with an on-demand menu. The m4RH usability evaluation assessed acceptability, feasibility, and potential behavioral impact [18]. The success of the project was evaluated based on the number of users interacting with the m4RH system, how users learned about the platform, and users reporting satisfaction with the program and its contents [18,19]. Similar to this ARMADILLO assessment, the m4RH assessment demonstrated the potential of reaching young people with factual and timely SRH information, including contraceptive facts. In addition, the (m4RH) assessment established that adolescents and young adults were the most frequent users of the system [18], with users appreciating the simplicity of the language used and the privacy that characterized the delivery of SRH information [19]. More assessments are needed to evaluate young people’s perceptions of the ease of use and acceptability of mHealth systems and their contents.

### mHealth Future Considerations

When a majority of participants successfully engaged with the ARMADILLO intervention, why choose to focus this paper on the usability pain points? The success and adoption of mHealth interventions largely depends on the targeted end users’ interaction with the technology and their belief that using the platform will benefit their health [4]. With regard to content development, the ARMADILLO study had a robust formative phase [25] that involved vetting SRH content with young people for comprehension, relevance, and tone. That said, during its implementation, a small minority of participants indicated that they struggled to understand the content. This speaks to the importance of understanding the full spectrum of health literacy among the targeted population, particularly when dealing with a population as heterogeneous as young people.

Issues related to the construction of the system itself (the *stop* feature, any confusion about the short code and progressing through the system) might possibly have been identified by conducting additional pre-RCT *real-world* pretest of the system. Others, including sustained frustration at not being able to obtain additional information, likely did not affect the objectives of the broader RCT but had implications for implementing similar services. One preferred feature of the ARMADILLO intervention was the instant gratification of immediately receiving a targeted message when requested. This is the natural strength of an on-demand system where the desired information comes when queried.

Sentiments about the desire for real-time feedback were observed with another digital study in Kenya [26]. This can happen in a few ways. One option is to have persons (peers or health experts) providing real-time responses to questions, as has been done in Mozambique’s Geracao Biz program [27]—this can, however, be resource intensive. Rapid improvements in artificial intelligence and its application to chatbots mean that users may soon be able to receive more *human* responses to real-time queries. Springster’s Big Sis is an interesting early example [25], although evaluation is needed. A less expensive option is to link users of on-demand or push systems to platforms with more flexibility and space for content (eg, websites) and hotlines. Integrating with already-existing systems avoids needless duplication. Creating robust and interoperable platforms ensures the success of mHealth initiatives in readiness for scale-up [28].

### Limitations

This study was not without limitations, one being that intervention participants not interviewed in this study might have different perspectives on the ARMADILLO platform compared with those reported in this paper. However, the random selection of intervention arm study participants and participants’ diversities in terms of age and gender strengthen our recruitment criteria by avoiding a biased selection. Second, respondents in this study might have given socially desirable responses by either overreporting the positives or underreporting of undesirable platform features. The consistency of our findings with other mHealth assessment studies supports the validity of this study.

### Conclusions

Findings from this study add evidence to the underexplored area of mHealth users’ experiences targeting young people. The need to consider end users throughout the mHealth development for optimum functionality to be achieved is key. This will not only create a sense of ownership but also give the mHealth initiative a practical approach while promoting its adoption through various media appealing to young people. With continued attention to digital health broadly and an entire new generation of delivery channels (from influencers to chatbots), program developers need to pay careful attention to understanding how users interact with a system, rather than make assumptions about *what works* when engaging with young people.

## Abbreviations

ARMADILLO: Adolescent/Youth Reproductive Mobile Access and Delivery Initiatives for Love and Life Outcome
CPR: contraceptive prevalence rate
IDI: in-depth interview
mHealth: mobile health
m4RH: Mobile for Reproductive Health
RCT: randomized controlled trial
SRH: sexual and reproductive health
WHO: World Health Organization
